# Association between gestational diabetes mellitus, maternal health and diet, and gut microbiota in mother-infant dyads

**DOI:** 10.1186/s12884-025-07584-2

**Published:** 2025-04-24

**Authors:** Isabelle Petitclerc, Julie Perron, Camille Dugas, Thomas Mayer, Frédéric Raymond, Vincenzo Di Marzo, Alain Veilleux, Julie Robitaille

**Affiliations:** 1 Centre de recherche Nutrition, Santé et Société (NUTRISS), Institute of Nutrition and Functional Foods (INAF), Université Laval, Quebec City, QC G1V 0A6 Canada; 2 School of Nutrition, Université Laval, Quebec City, QC G1V 0A6 Canada; 3 Canada Research Excellence Chair in the Microbiome-Endocannabinoidome Axis in Metabolic Health (CERC-MEND), Université Laval, Quebec City, QC G1V 0A6 Canada; 4 Centre de recherche de l’Institut universitaire de cardiologie et de pneumologie de Québec (IUCPQ), Université Laval, Quebec City, QC G1V 4G5 Canada

**Keywords:** Gestational diabetes mellitus, Gut microbiota, Cardiometabolic profile, Maternal diet, Mother-infant health

## Abstract

**Background:**

Gestational diabetes mellitus (GDM) increasingly affects women and predisposes both mothers and their infants to short- and long-term health consequences. Emerging research links GDM to maternal gut microbiota dysbiosis. However, the impact of GDM on the infant gut microbiota remains unclear. This cross-sectional study aims to explore potential associations between GDM and the gut microbiota in mothers and their infants, as well as correlations between maternal diet, cardiometabolic profile, and gut microbiota composition.

**Methods:**

Gut microbiota taxonomic composition was characterized by 16S rRNA gene sequencing on fecal samples collected at 2 months postpartum from 28 mothers, including 17 with (GDM+) and 11 without (GDM-) GDM, as well as 30 infants, 17 GDM + and 13 GDM-. Variations in overall composition and specific taxa between GDM + and GDM- were assessed. Correlations between maternal cardiometabolic profile, dietary intakes, and taxa were performed.

**Results:**

GDM was associated with the overall composition of gut microbiota between GDM + and GDM- in the maternal group, but not in infants. No statistically significant difference in alpha diversity between groups was found in either mothers or infants. However, 14 taxa showed significantly different abundance between GDM + and GDM- mothers, and 4 taxa differed in infants. Specific taxa at the family rank were correlated with maternal dietary and cardiometabolic variables in both mothers and infants.

**Conclusions:**

GDM exposition was associated with gut microbiota composition in both mothers and infants at two months postpartum. This study enhances our understanding of how maternal health could be linked with the gut microbiota of mothers and their infants.

**Trial registration:**

NCT02872402 (2016-08-04, https://clinicaltrials.gov/study/NCT02872402?term=NCT02872402&rank=1) and NCT04263675 (2020-02-07, https://clinicaltrials.gov/study/NCT04263675?term=NCT04263675&rank=1).

## Background

Gestational diabetes mellitus (GDM) is the onset of glucose intolerance first occurring during pregnancy [1]. It is well established that GDM predisposes mothers and their offspring to short- and long-term health consequences [2–5]. For instance, mothers who develop GDM, along with their children, are more likely to suffer from cardiovascular diseases such as type 2 diabetes later in life [6–10]. Additionally, GDM increases the risk of obesity in their offspring [10]. The incidence of GDM is rising globally due to factors such as advanced maternal age and increased rates of obesity, which are now more prevalent among pregnant women [11]. In 2021, the global prevalence of GDM was approximately 14% [12]. Therefore, developing effective prenatal and postnatal interventions is a public health priority, among others, to break the vicious intergenerational cycle of obesity and diabetes [13, 14]. Implementing interventions in the first 1000 days of life is particularly important, as The Developmental Origins of Health and Disease (DOHaD) concept highlights this period as an opportunity to reverse adverse prenatal exposure due to its high plasticity [15]. Indeed, it is now well established that early life interventions could prevent noncommunicable diseases [16]. Nevertheless, at this time, there is a lack of postnatal strategies to prevent complications among children exposed to GDM [17].

In recent years, there has been significant progress in our understanding of the microbiota and its association with disease pathogenesis [18]. For instance, gut microbiota dysbiosis is now recognized as a factor contributing to the pathophysiology of both type 2 diabetes [19, 20] and obesity [21]. Therefore, it is of great interest to study the associations between GDM and gut microbiota composition. GDM has already been associated with alterations in the mother’s gut microbiota composition during and after pregnancy [22–24].

Most factors shaping the infant’s microbiota in early life, including delivery mode, antibiotic use, gestational age, maternal diet, and feeding mode, are tied to the mother’s gut microbiota [25, 26]. Studies also indicated that the initial neonatal microbiota is shaped by the mother’s health status, such as diabetes [27, 28]. However, while some studies have also shown that the gut microbiota of infants exposed to GDM differed from that of infants born to healthy mothers [29], the full extent of this impact and its implications for child health are not yet fully understood [23, 30]. Likewise, the mechanisms explaining how GDM can alter the gut microbiota of mother and infant dyads remain unclear [29]. As dysbiotic neonatal microbiota is linked with diseases in childhood and adulthood, investigating the impact of GDM on the microbiota may provide valuable insights into the mechanisms underlying the predisposition of children exposed to GDM to various complications [31]. This may help to develop effective intervention strategies for this high-risk population.

Therefore, the first objective of this exploratory study was to investigate potential associations between GDM and the gut microbiota in mothers and their infants. The second objective was to explore whether factors such as maternal diet and cardiometabolic profile correlate with changes in gut microbiota linked to GDM. We hypothesize that GDM is associated with the gut microbiota of mothers and infants. Additionally, we hypothesize that specific cardiometabolic variables related to diabetes, as well as dietary components, correlate with the gut microbiota of both mothers and infants.

## Methods

### Study cohort

This study is a secondary analysis of data from a previous randomized controlled trial (RCT). Specifically, women with gestational diabetes (GDM+) were enrolled in a pilot RCT to assess the feasibility and acceptability of a lifestyle intervention starting at 2 months postpartum and continuing until 18 months postpartum (clinical trial NCT02872402). The target of this intervention was to prevent type 2 diabetes in women who had developed GDM during their pregnancy. Pregnant women followed at one of the two hospitals in Quebec City (Canada) with a neonatal care unit and who were diagnosed with GDM using the 2013 clinical guidelines from the Canadian Diabetes Association (CDA) were invited to participate in this clinical study. In 2013, CDA’s “preferred approach” to screening consisted of a 50 g glucose challenge test (GCT) at 24 to 28 weeks followed by a 75 g oral glucose tolerance test (OGTT) if plasma glucose at one hour is ≥ 7.8 mmol/L [32]. Additionally, emails were sent to the Université Laval community to invite eligible women to participate in this study. Inclusion criteria included being ≥ 18 years old, with a body mass index (BMI) ≥ 18.5 kg/m^2^, proficiency in French, and a confirmed GDM diagnosis. Exclusion criteria were multiple pregnancies, a history of type 1 or type 2 diabetes, preterm delivery (< 37 weeks), prior bariatric surgery, and any plans for pregnancy within the next year. Recruitment spanned from January 2017 to September 2019. GDM + mothers were treated for their GDM according to Diabetes Canada Guidelines [11]. Mothers without a history of gestational diabetes (GDM-) who met the same exclusion and inclusion criteria, except for having no previous history of GDM were recruited at 2-month post-delivery through emails sent to the Université Laval community and social media posts from March to September 2020 (clinical trial NCT04263675). From the 62 mother-infant dyads initially recruited in the RCT, only those with fecal samples collected at 2 months postpartum (at baseline) were included in this study, resulting in a total of 28 mothers (17 GDM + and 11 GDM-) and 30 infants (17 GDM + and 13 GDM-). As the primary objective of the RCT was not to analyze microbiota, fecal sample collection was optional. Of these, we had a total of 27 mother-infant dyads, as one mother provided only her fecal sample and three provided only their infant’s sample. This study was conducted according to the guidelines laid down in the Declaration of Helsinki and all procedures involving human subjects were approved by the Centre Hospitalier Universitaire de Québec Ethics Committee (2017–3225 and 2020–5075). Written informed consent was obtained from all subjects.

### Participants’ characteristics

Mothers completed self-administered questionnaires covering sociodemographic characteristics, infant feeding practices, and antenatal data. Only data collected at the baseline visit, at 2 months postpartum, were used for the current study. Mothers were weighted on a calibrated balance (Tanita BC-418) to the nearest 0.1 kg, and their height was measured to the nearest 0.1 centimeter using a stadiometer. BMI was then calculated (kg/m^2^). Body composition was measured by dual-energy X-ray absorptiometry scanner (DEXA, GE Healthcare Lunar; Madison, WI, USA). Fat mass percentages, including gynoid and android fat mass, were analyzed as described previously [5]. Women self-reported their pre-pregnancy BMI as well as pregnancy weight gain. A fasting blood sample was collected before performing a 75 g 2-hour oral glucose tolerance test. Glycemia and insulinemia levels at 0 and 2 hours were measured enzymatically and by electrochemiluminescence, respectively [33, 34]. The Homeostasis Model Assessment of Insulin Resistance (HOMA-IR) was calculated using the formula: (fasting insulinemia (mIU/L)·x fasting glycemia (mmol/L)/22.5) [35]. Glycated hemoglobin (HbA1c), total cholesterol, HDL and LDL cholesterol, and triglycerides were also measured in the blood sample.

### Dietary assessment

Maternal dietary data at 2 months postpartum were obtained by a validated, self-administered web-based food frequency questionnaire [36].

### Sample collection

Fecal samples for both mothers and infants were collected at home by mothers near the 2-month postpartum visit, stored in sterile tubes, and immediately stored in freezers at -20^o^C. Mothers were also requested to precisely document the timing of sample collection and freezing. On the morning of the appointment, mothers had to bring the frozen samples in separate transport bags with ice packs. The samples were then frozen at -80^o^C until further DNA extraction.

### 16 S rRNA gene sequencing

Stool bacterial DNA was extracted using QIAamp Fast DNA Stool Mini Kit (Qiagen, CA, USA). The V3-V4 region of the 16S rRNA gene was amplified using the primers S-D-Bact-0341-b-S-17(5’- TCG TCG GCA GCG TCA GAT GTG TAT AAG AGA CAG CCT ACG GGN GGC WGC AG- 3’), and S-D-Bact-0785-a-A-21 (5’-GTC TCG TGG GCT CGG AGA TGT GTA TAA GAG ACA GGA CTA CHV GGG TAT CTA ATC C- 3’) (IDT, IA, USA) as previously described [37]. Briefly, libraries were purified using magnetic beads (AMPure XP, Beckman Coulter, CA, USA), and their quality was assessed using the QIAxcel Advances System (QIAGEN, Hilden, Germany). MiSeq platform (Illumina, CA, USA) was used to perform high-throughput sequencing (2- by 300-bp paired-end). Sequences were processed with the Dada2 package (Version 1.10.1) [38], and bacterial taxa were identified using the Silva v132 reference database [39]. Sequences present in fewer than 5 samples were filtered out. For statistical analysis, bacterial relative abundance was normalized using Cumulative Sum Scaling (CSS, MetagenomeSeq R package, version 1.40.0) [40].

### Statistical analyses

All statistical analyses were performed on R Studio Software (R version 4.2.2). Infants’ and mothers’ characteristics were compared between GDM + and GDM- using Fisher’s exact test and Independent Samples t-test where appropriate. Various tests were performed to investigate associations between GDM and gut microbiota composition. A linear model was used to identify the taxa that best explain the variance in gut microbiota between the two groups. Alpha diversity metrics were calculated using the Phyloseq package (version 1.42.0). Simpson and Chao 1 diversity indices were computed for each sample. Chao 1 assesses richness (the total number of species in a community), while the Simpson index considers both richness and evenness (the relative abundance of species in a community) [41]. Correlations of mothers’ dietary components and cardiometabolic profiles with infants’ and mothers’ gut microbiota composition at family levels were computed using Spearman’s rank correlations. While our initial analysis encompassed a variety of food groups, including fish, nuts, animal and plant-based proteins, fruits, and vegetables, we focused the presented results on the primary food groups and dietary components that have been most strongly associated with gut microbiota composition [42–47] and those for which we identified noteworthy correlations. By doing so, we ensure that the findings are presented clearly, highlighting their significance. Multiple factor analysis (MFA), a dimensionality reduction method used to analyze several groups of variables collected from the same set of observations [48], was performed using the FactoMineR R package (version 2.8) and the Factoextra R package (version 1.0.7). In MFA, the dimensions (the axes) represent combinations of the variables that best separate the samples, with the first and second dimensions explaining the greatest variability. Three separate MFA models were constructed: one encompassing all samples, one for infants, and another for mothers. These variables included infant-specific factors (sex, age at sampling, and feeding type), maternal dietary variable (daily intake of grains, vegetables, milk products, and total dietary fiber), maternal cardiometabolic health variable (GDM status, type of GDM treatment, BMI, HbA1c, HOMA-IR, and percentage of android fat), and gut microbiota family [CCS-normalized relative abundance of bacterial families in at least 10% of individuals, *n* = 40)], and phyla [CCS-normalized relative abundance of bacterial families in at least 10% of individuals, *n* = 6)]. Permutational multivariate analysis of variance (PERMANOVA) was used to compare parameters between GDM groups. Euclidean distances were calculated using coordinates from the MFA results, using the stats package (version 4.2.2) to assess beta diversity between samples. Generalized linear models (GLM) were then used to identify which parameters were associated with the distances between samples.

## Results

### Mothers and infants’ characteristics

The characteristics of the study participants are summarized in Table 1. Prepregnancy BMI, maternal age, as well as 2-hour insulinemia and glycemia were significantly higher among mothers from the GDM + group (*p* < 0.05), whereas gestational age at birth was significantly lower in this group. Among infants, age at the 2-month baseline visit was significantly higher in the GDM + group (*p* < 0.05).

**Table 1:** Characteristics of mothers and infants

Mothers			
*Characteristics*	*Total, n = 28*	*GDM-, n = 11*	*GDM+, n = 17*
**Demographic and antenatal data**			
Ethnicity			
Caucasian	28 (100)	11 (100)	17(100)
Age (years)	32.9 ± 3.6*	31.2 ± 3.0*	33.9 ± 3.7*
Prepregnancy BMI (kg/m^2^)	30.4 ± 7.5*	26.5 ± 6.4*	32.5 ± 7.3*
GDM treatment			
Insulin + diet	7(25)	0(0)	7(41.18)
Hypoglycemic agent + diet	2(7.1)	0(0)	2(11.8)
Diet	7(25.0)	0(0)	7(41.2)
None	12(42.9)	11(100)	1(5.9)
Cesarean section			
Yes	2(7.1)	1(9.1)	1(5.9)
No	26(92.9)	10(91.0)	16(94.1)
Pregnancy weight gain (kg)	10.3 ± 4.7	11.6 ± 4.9	9.6 ± 4.5
Gestational age (weeks)	39.0 ± 1.2*	39.7 ± 0.9*	38.5 ± 1.1*
**Metabolic at 2 months post postpartum**			
BMI (kg/m^2^)	30.9 ± 6.8	28.3 ± 6.2	32.5 ± 6.9
BMI groups			
Normal	4(14.3)	3(27.3)	1(5.9)
Overweight	10(50)	4(36.4)	6(35.3)
Obese	14(35.7)	4(36.4)	10(58.8)
Fat mass (%)	42.1 ± 7.4	38.9 ± 8.6	44.2 ± 5.9
Android fat (%)	45.1 ± 9.8	41.0 ± 12.7	47.8 ± 6.6
Gynoid fat (%)	47.1 ± 7.5	44.0 ± 7.9	49.1 ± 6.7
Fasting glycemia (mmol/L)	5.0 ± 0.4	4.9 ± 0.3	5.0 ± 0.4
2-Hour glycemia (mmol/L)	5.5 ± 1.1*	4.9 ± 0.8*	5.9 ± 1.2*
Fasting insulinemia (pmol/L)	56.0 ± 34.4	59.5 ± 23.4	53.6 ± 478.0
2-Hour insulinemia (pmol/L)	282.0 ± 174.5*	197.3 ± 96.2*	329.6 ± 192.5*
HbA1c (%)	5.2 ± 0.3	5.2 ± 0.3	5.2 ± 0.3
HOMA-IR	1.81 ± 1.16	1.90 ± 1.56	1.75 ± 0.85
**Infant feeding practices**			
Any Breastfeeding			
Yes	27(96.4)	11(100)	16(94.1)
No	1(3.6)	0(0)	1(5.9)
Exclusive breastfeeding			
Yes	23(82.1)	11(100)	12(70.6)
No	5(17.9)	0(0)	5(29.4)
**Infants**			
***Characteristics***	***Total, n = 30***	***GDM-, n = 13***	***GDM+, n = 17***
Sex			
Male	14(46.7)	4(30.8)	10(58.8)
Female	16(53.3)	9(69.2)	7(41.2)
Age (days)	82.0 ± 12.9*	72.8 ± 10.7*	89.3 ± 9.6*
Birth weight (kg)	3.39 ± 0.37	3.40 ± 0.45	3.37 ± 0.31
Weight at 2 months (kg)	5.11 ± 0.80	5.09 ± 0.55	5.12 ± 0.10
Birth weight-for-age z-score	0.19 ± 0.79	0.25 ± 0.94	0.14 ± 0.68
Weight-for-age z-score at 2 months	-0.20 ± 0.90	-0.20 ± 0.84	-0.20 ± 0.97
Change in weight-for-age z-score from birth to 2 months	-0.39 ± 0.84	-0.46 ± 0.81	-0.34 ± 0.89

* *p*-value < 0.05

### Maternal and infant gut microbiota composition

**Fig. 1:**
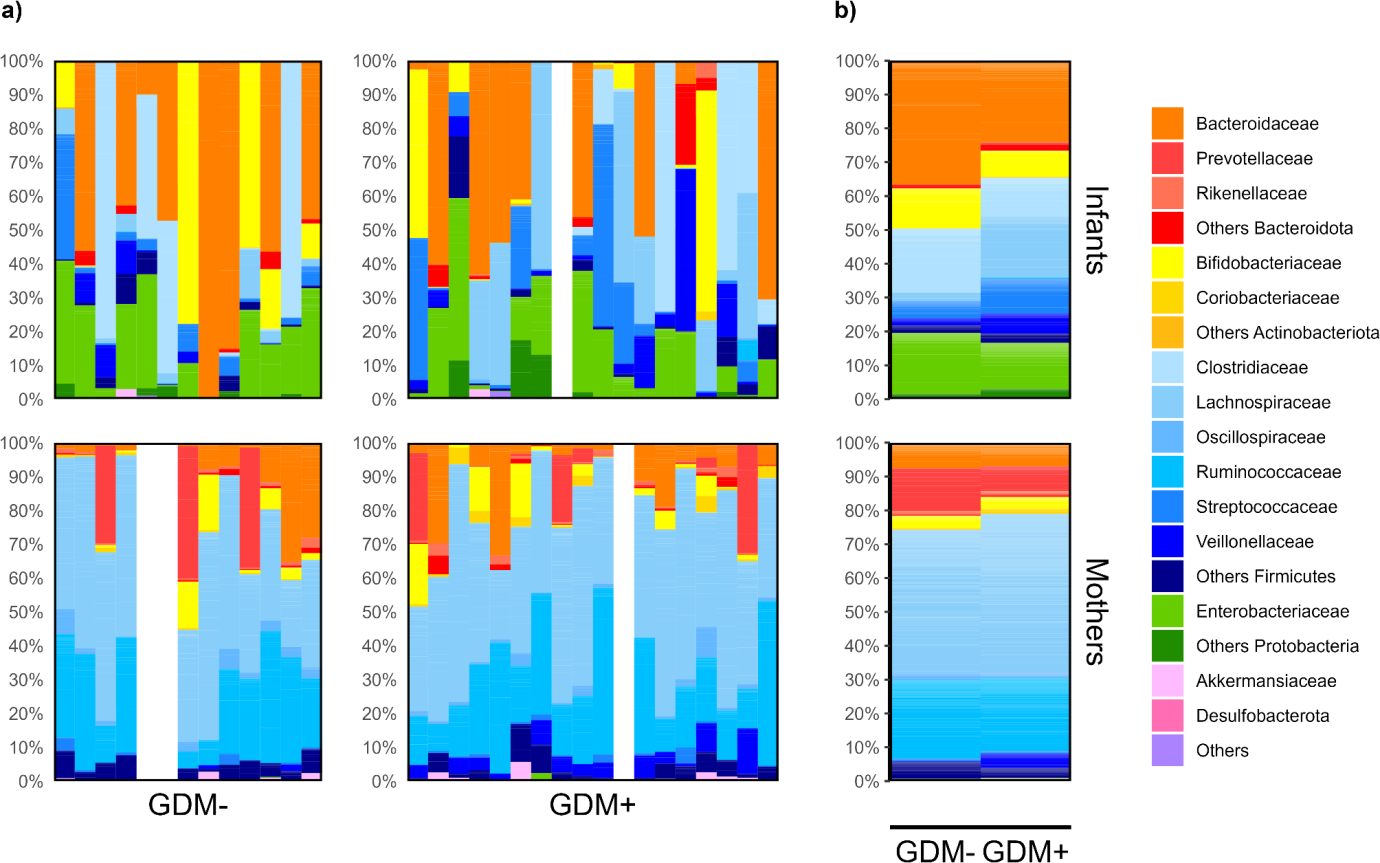
Relative abundance of gut microbiota taxa at the family level between GDM + and GDM- groups. (**a**) Individual samples; (**b**) GDM- and GDM + groups in mothers and infants

**Fig. 2:**
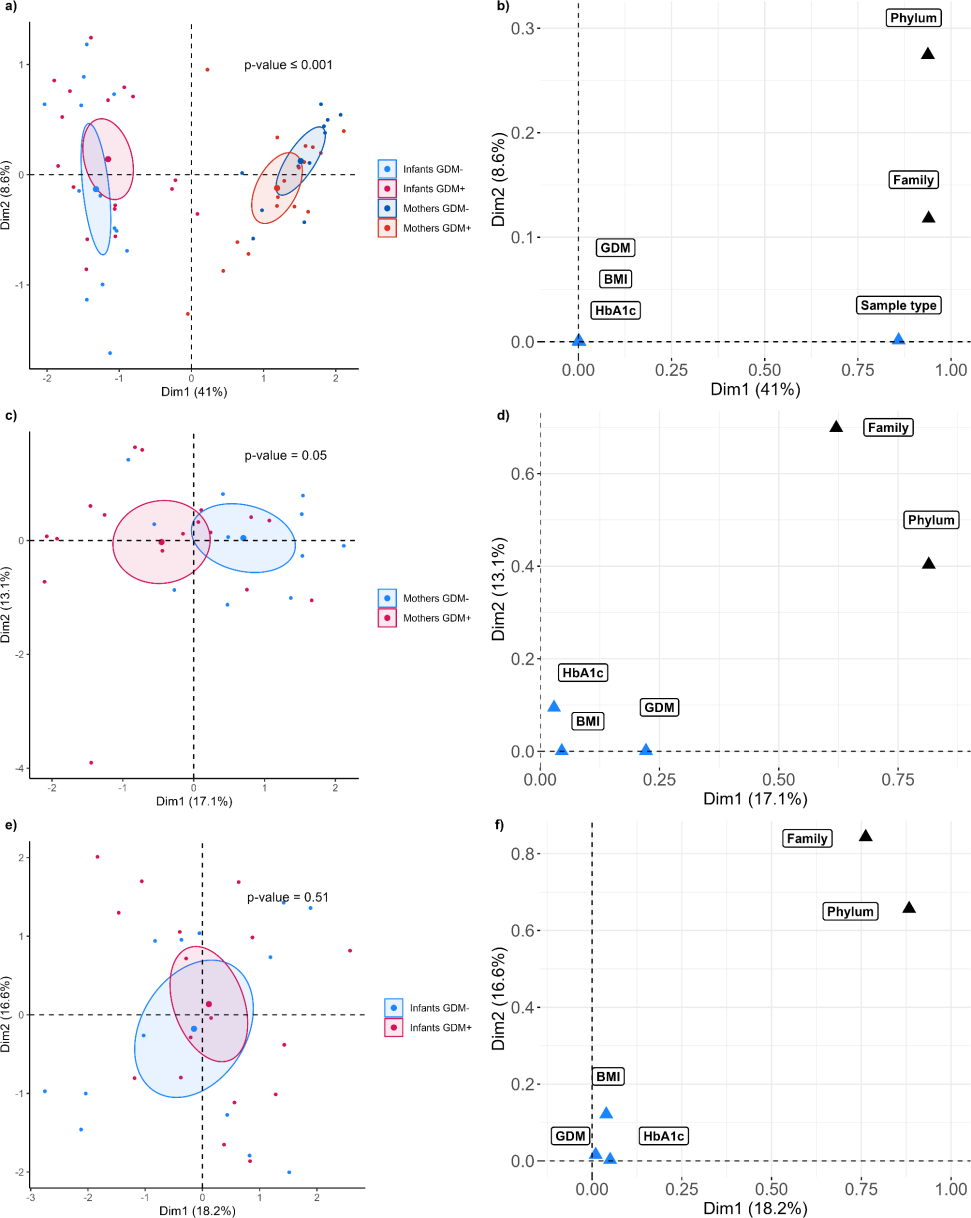
Multiple factor analysis (MFA) modeling of gut microbiota for dimensions 1 and 2. (**a**) Comparison between infants and mothers and between GDM- and GDM+, (**c**) Comparison between GDM- and GDM + mothers, (**d**) Comparison between GDM- and GDM + infants. Ellipses represent standard deviations from the mean center of each group. Graph of variable contributions to dimensions 1 and 2: (**b**) the entire sample, (**d**) mothers, and (**f**) infants

**Fig. 3:**
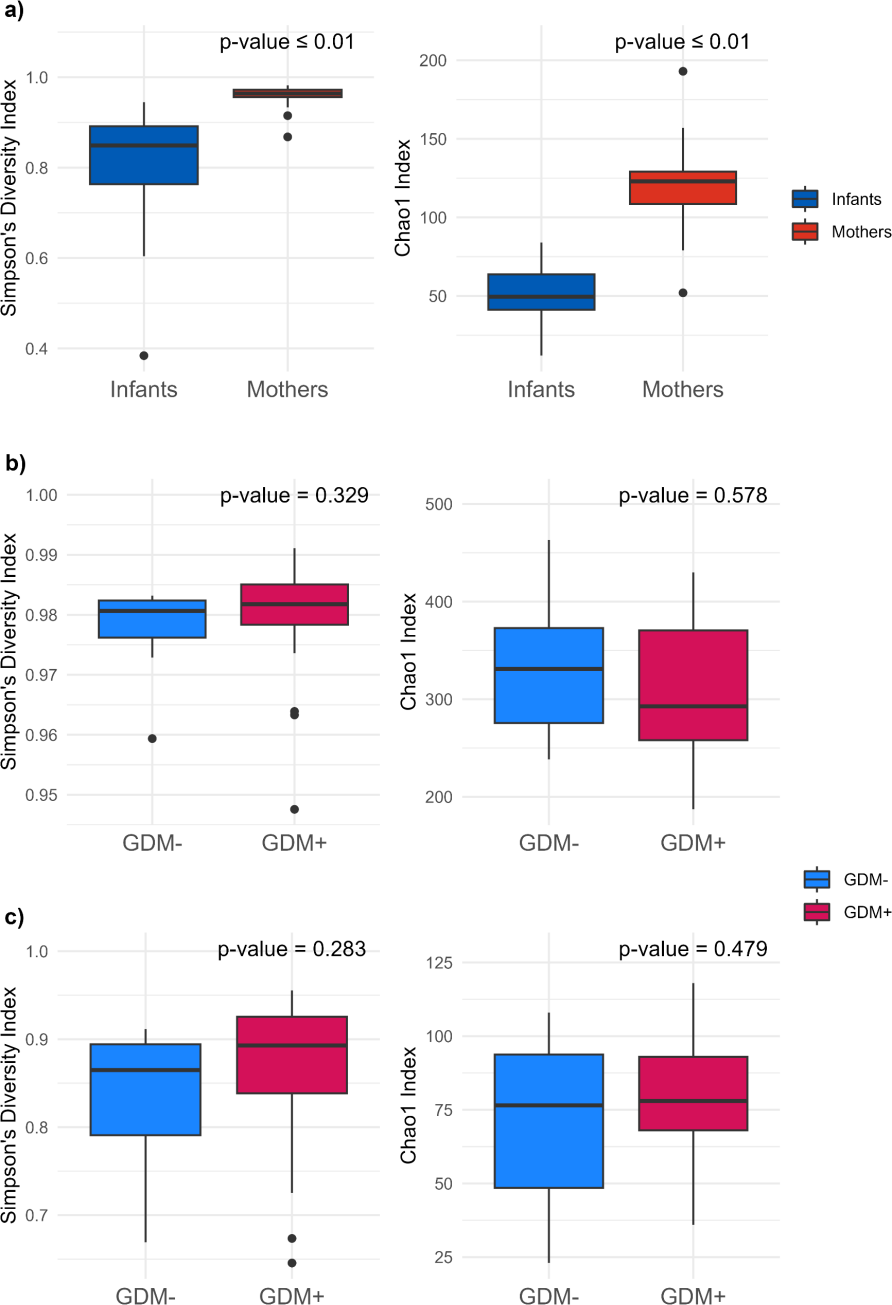
Gut microbiota alpha diversity indices measuring evenness (Simpson index) and richness (Chao 1 index). (**a**) Comparison between infants and mothers; (**b**) Comparison between GDM- and GDM + mothers; (**c**) Comparison between GDM- and GDM + infants. p values are from Wilcoxon tests

While a large interindividual variability was observed in the gut microbiota composition of our sample, as shown by the histograms illustrating each participant’s taxa relative abundance, (Fig. 1a), its composition differed significantly between infants and mothers (PERMANOVA, *p* ≤ 0.001) (Fig. 2a). In mothers, families in the Firmicutes phylum were predominant (76.7%), followed by those in the Bacteroidota phylum (17.1%). Families in the Actinobacteriota (5.2%) and Proteobacteria (0.1%) phyla were of low abundance. Among infants, families in the Firmicutes phylum accounted for a large proportion of the overall microbiota (41.3%). In comparison to mothers, a higher relative abundance of families in the Bacteroidota (30.0%), Actinobacteriota (10.5%), and Proteobacteria (17.8%) phyla was observed in infants’ gut microbiota (Fig. 1b). Furthermore, the Simpson and Chao 1 alpha diversity indices were significantly higher in the mothers’ gut microbiota compared to that of the infants (Wilcoxon, *p* ≤ 0.01) (Fig. 3a).

### Gut microbiota composition according to GDM status

No statistical differences were found in either the Chao 1 or Simpson diversity indices between GDM + and GDM- groups, both in mothers and infants (Fig. 3b and c). However, confidence interval ellipses for GDM + and GDM- mothers were mostly separated on MFA graphs and the PERMANOVA analysis detected a significant difference between groups (*p* = 0.05), showing that the overall gut microbiota composition of mothers differed according to their GDM status (Fig. 2c). GDM appears to explain a part of the first dimension, accounting for 17.1% of the variance, while other variables often associated with GDM such as maternal BMI and HbA1c did not (Fig. 2d). Indeed, a GLM revealed that GDM significantly predicted the first dimension (*p* = 0.01), even after adjusting for maternal BMI and HbA1c levels (*p* = 0.02). In contrast, neither BMI nor HbA1c were significantly associated with this dimension (*p* = 0.99 and *p* = 0.47, respectively). For the infants, gut microbiota composition between GDM + and GDM- groups was similar based on the MFA confidence interval ellipses and PERMANOVA analysis (*p* = 0.51), suggesting that maternal GDM status was not associated with notable differences in the overall infant microbiota composition (Fig. 2e). The GDM status did not significantly explain either the first dimension, accounting for 18.2% of the variance, or the second dimension, explaining 16.6% of the variance (GLM, respectively *p* = 0.5 and *p* = 0.6) (Fig. 2f). However, a GLM revealed a near-significant association between the variances explained in the second dimension and maternal BMI (*p* = 0.06). This suggests that while GDM is not a major factor associated with the composition of the infant microbiota, other maternal variables, such as adiposity, may be associated with differences in its composition.

**Fig. 4:**
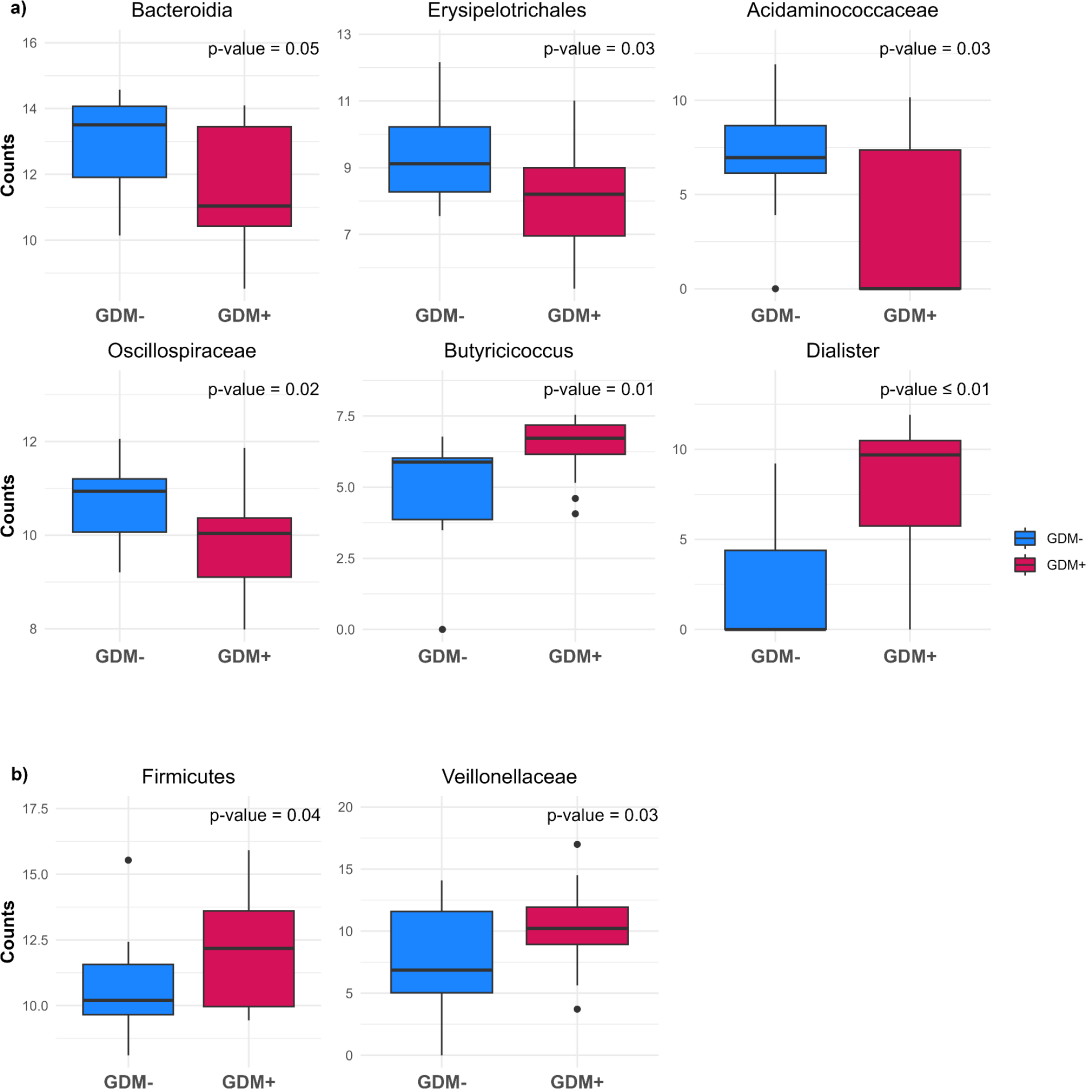
Changes in normalized bacterial counts between GDM- and GDM + groups. (**a**) Mothers; (**b**) Infants. p-values are from Wilcoxon tests

When each taxon was analyzed according to GDM status, we observed significant differences between the two groups. GDM + mothers exhibited significantly higher levels of the genus *Dialister*, along with its corresponding family and order. Similarly, the genus *Butyricicoccus* and its corresponding family were more abundant in GDM + mothers (*p* ≤ 0.05). Conversely, the family *Oscillospiraceae* and the order Erysipelotrichales with their corresponding class, the family *Acidaminococcaceae* and its corresponding order, and the class Bacteroidia with its phylum were lower in GDM + mothers (*p* ≤ 0.05) (Fig. 4a). In GDM + infants, taxa within the Firmicutes phylum were significantly increased, along with *Veillonellaceae*, its corresponding class, and order (*p* < 0.05) (Fig. 4b). Analyses were adjusted for potential confounding variables (maternal BMI and maternal HbA1c), and similar results were observed (not shown).

### Associations between maternal diet and cardiometabolic profile with the infant and mother gut microbiota composition

**Fig. 5:**
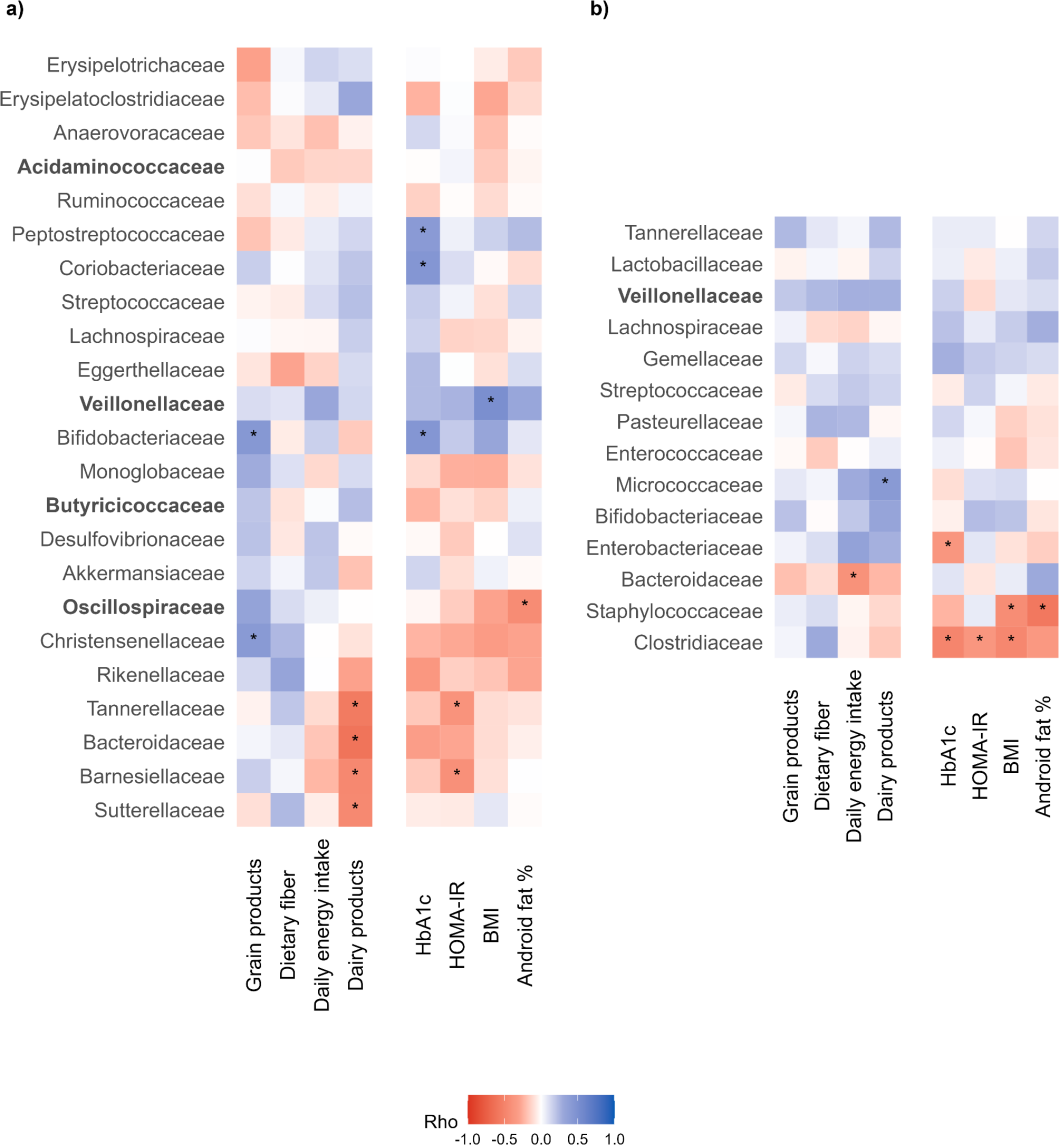
Correlation heatmap of maternal cardiometabolic profile and diet with family level gut microbiota taxa. (**a**) Mothers and (**b**) Infants. Relative abundance of taxa in bold are significantly different between GDM groups. Stars (*) indicate significant correlations. The correlation coefficients were calculated using Spearman’s rank correlation

To deepen our understanding of how maternal GDM might be associated with both infant and mother gut microbiota, we aimed to examine which maternal factors are associated with gut bacterial composition at the family taxonomic rank. In mothers, two of the four families that showed significantly different relative abundance between GDM + and GDM- mothers were correlated with maternal characteristics. Indeed, *Veillonellaceae* relative abundance was positively correlated with BMI while *Oscillospiraceae* relative abundance was negatively correlated with android fat percentage. We also observed that HbA1c was significantly positively correlated with *Peptostreptococcaceae*, *Coriobacteriaceae*, and *Bifidobacteriaceae*, while HOMA-IR was negatively correlated with *Tannerellaceae* and *Barnesiellaceae* (Fig. 5a). Maternal grain product consumption was positively correlated with the abundance of *Bifidobacteriaceae* and *Christensenellaceae*, while dairy product consumption was negatively correlated with *Tannerellaceae*, *Bacteroidaceae*, *Barnesiellaceae*, and *Sutterellaceae*. Among infant microbiota, we observed that maternal HOMA-IR, HbA1c, and BMI were associated with *Clostridiaceae* while maternal android fat percentage and BMI were negatively associated with *Staphylococcaceae*. Finally, no statistically significant correlation was found with *Veillonellaceae*, the only family for which the relative abundance significantly differed between GDM + and GDM- infants (Fig. 5b).

### Similarity between mother-infant dyads

**Fig. 6:**
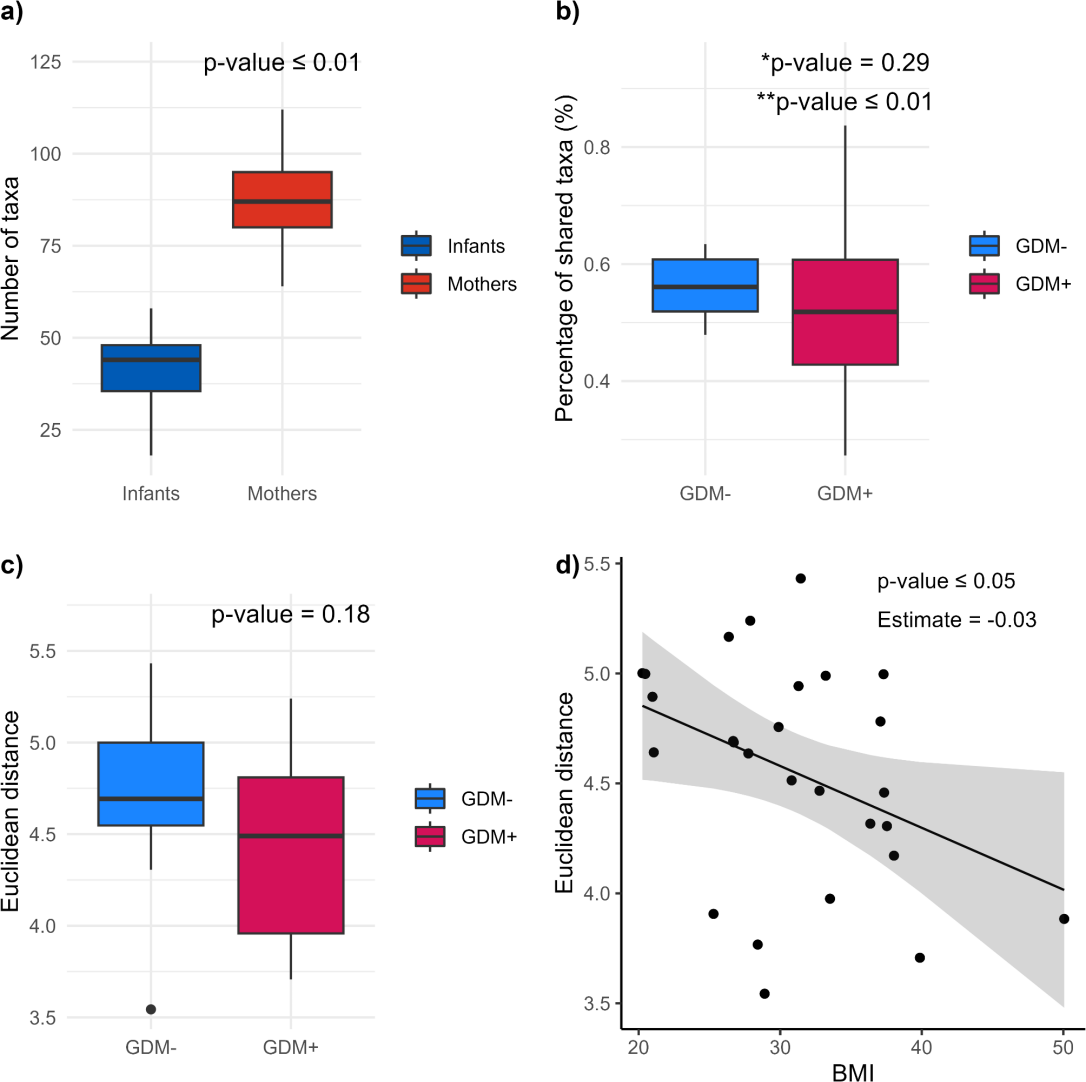
Similarity in gut microbiota between mother-infant dyads. (**a**) Number of taxa per sample, categorized by sample type; (**b**) Percentage of taxa commonly shared within mother-infant dyads, grouped by GDM exposure. *p-value for Wilcoxon test; **p-value for Levene’s test. (**c**) Euclidean distance between mother-infant dyads, grouped by GDM exposure. (**d**) Euclidean distance between mother-infant dyads plotted against maternal BMI. All other p-values are from the Wilcoxon test

We examined the similarity between mother-infant dyads. For this analysis, we excluded unpaired samples, resulting in a total of 27 pairs (1 mother and 3 infants were removed). Mothers harbored a greater diversity compared to infants, with an average of 88 taxa compared to an average of 42 in infants (Wilcoxon, *p* ≤ 0.01) (Fig. 6a). On average, 22 taxa were shared between pairs, which corresponds to about 54% of the taxa in infants being common with their respective mothers. No significant differences were found in the percentage of shared taxa and the distance within each dyad between GDM + and GDM- groups (*p*> 0.05). However, the boxplot of percentages of shared taxa between dyads (Fig. 6b) revealed that values were more scattered in the GDM + group, this significant difference in variability being confirmed by a Levene’s test (*p* ≤ 0.01). Specifically, the GDM + group contained dyads with both the highest and the lowest percentages of shared taxa. Although no direct association was found between GDM and the percentage of shared taxa, including BMI as a covariate revealed that adiposity may explain much of the variance in the percentage of shared taxa within dyads. Indeed, while no significant association was found with GDM status or HbA1c levels (Fig. 6c), we observed that BMI was negatively associated with Euclidean distances of gut microbiota relative abundances within dyads (GLM Estimate = -0.03, *p* ≤ 0.05, Fig. 6d)).

## Discussion

In this study, we investigated whether exposure to GDM during pregnancy is associated with the gut microbiota composition of mothers and infants at 2 months postpartum. We found that GDM was associated with the relative abundance of certain taxa in both mothers and infants. The overall composition of the maternal gut microbiota was associated with GDM exposure, independent of maternal BMI. However, no clear separation between the overall gut microbiota of infants exposed or not to GDM was observed.

Results of our study show that the mother gut microbiota composition was associated with GDM status, consistent with previous literature. Indeed, a recent systematic review also found that all included studies reported differences in postpartum gut microbiota composition between mothers with GDM and controls [49]. We also observed that GDM was significantly associated with gut microbiota composition, even after adjusting for BMI. This finding is of particular interest as increased BMI is associated with GDM [50], and being overweight has been linked to variations in gut microbiota in pregnant women [51] as well as in women in the postpartum period [52]. This finding is also consistent with the report of Crusell et al. who also observed differences in the gut microbiota of postpartum GDM + mothers even after BMI adjustment [24].

Moreover, we observed specific differences in the relative abundance of taxa between GDM + and GDM- maternal gut microbiota such as an increase of *Dialister* and *Butyricicoccus* and a decrease of Bacteroidia, Erysipelotrichales, *Acidaminococcaceae*, and *Oscillospiraceae* in GDM + mothers. These specific taxa have not previously been reported to be altered in GDM, although results from previous studies have been inconsistent [49, 53]. Gut microbiota composition can be influenced by various factors, such as the timing of postpartum sampling, geographic location, diet, and medication, which limits comparisons between studies [54, 55].

Although four taxa were significantly associated with GDM status, these associations did not result in significant differences in the overall gut microbiota composition, as no notable distinction was observed between the GDM + and GDM- groups in infants. The abundance of Firmicutes and *Veillonellaceae* was significantly higher in the infant GDM + group. Other studies have highlighted significant differences in other taxa between GDM + and GDM- infants in different studies [56–65]. Similar to our results, Chen et al. analyzed meconium microbiota in the context of GDM and found that the GDM + group exhibited a significantly higher relative abundance of Firmicutes [57]. Increased Firmicutes abundance has been linked with weight gain in infants, as noted by Indiani et al., who observed a positive correlation between an increase in BMI of infants between 9 and 18 months and the Firmicutes phylum [66]. When looking specifically at taxa at the family rank, *Veillonellaceae* was the only one that showed a significant increase in the GDM + group. This result contrasts with data from Wiinblad Crusell et al., who observed an enrichment of *Veillonellaceae* in the gut microbiota of 1-week old newborns without in utero exposure to GDM [56]. This discrepancy may be explained by the increased abundance of *Veillonellaceae* among both GDM + infants and mothers in our study, as well as differences in study populations. Participants included in this study were from Canada, while those in the study by Crusell et al. were from Denmark, leading to differences such as variations in dietary patterns. It is indeed established that geographic origin and diet are significant factors influencing gut microbiota composition [67].

*Veillonellaceae* has previously been found to have a higher abundance in insulin-resistant adults, as noted by Naderpoor et al. [68], and Murri and al. observed a significant increase in *Veillonella* (a genus in the *Veillonellaceae* family) in children with type 1 diabetes [69]. Additionally, a systematic review by Que et al. found a consistent trend of increased *Veillonellaceae* and Firmicutes in participants with type 2 diabetes compared to healthy controls [70]. Fecal *Veillonellaceae* have also been suggested to be positively correlated with endocannabinoid-like *N*-acylethanolamines with a strong role in the control of inflammation and glucose and lipid metabolism, and to be stimulated by these mediators [71], some of which we have previously reported being increased in the milk of the mothers of the same cohort used in the present study [72]. *N*-acylethanolamines, if passed with milk from mother to child [73, 74], could represent a molecular link: (1) between the dysbiotic gut microbiota of the mother and its negative effects on the metabolic state of the infant, even beyond the infant gut microbiota, or (2) between the effects of the mother gut microbiota on that of the infant even beyond the direct transmission of taxa from the former to the latter.

Indeed, in the current study, maternal characteristics were found to be associated with the gut microbiota of both mothers and infants. Specifically, dairy and grain product consumption were associated with maternal microbiota. This is consistent with previous research [42–45]. We also observed that the maternal cardiometabolic profile was correlated with specific taxa in the mother microbiota similar to previous studies [75–77]. Additionally, maternal variables were correlated with some taxa in the infant gut microbiota. Ponzo et al. similarly reported some correlation between maternal parameters and infants in the context of GDM [58]. This could be explained by the vertical transmission of bacteria from the mother’s body and breastmilk to her infant [78].

No statistical differences were found in alpha diversity between the GDM + and GDM- groups in either infants or mothers. A lower alpha diversity is a characteristic of dysbiotic microbiota, which is associated with various chronic diseases [79–81]. Dysbiosis is an imbalance in the gut microbiota which is commonly characterized by a decrease in beneficial bacteria, an increase in pathogenic microorganisms, and a reduced microbial diversity [82]. However, the literature on GDM impact on alpha diversity is inconclusive [49]. Few studies have focused on postpartum maternal microbiota in the context of GDM [49, 53], and those that did generally found no significant differences in alpha diversity between exposed and unexposed groups [24, 83, 84]. The complexity of the gut microbiota ecosystem, shaped by multiple factors, such as diet, genetics, and environment, may outweigh the effect of GDM on gut microbiota diversity [54]. Studies on the infant microbiota in a GDM context are inconsistent. Some found that alpha diversity tends to be lower in infants exposed to GDM [57, 58], while others, like us, found no significant differences [59, 60]. Crusell et al. found a lower richness in newborns exposed to GDM, and this difference disappeared by 6–18 months as richness increased [56]. Therefore, the association between GDM exposure and the overall infant gut microbiota diversity may be most noticeable on the first days, gradually becoming less apparent as other strong environmental factors shape the infant microbiota over time [85, 86]. This highlights the importance of longitudinal studies in understanding the impact of GDM on infant microbiota.

However, we did find that maternal BMI was significantly associated with the similarity of gut microbiota between infants and mothers. Specifically, a higher BMI was associated with more shared species within a mother-child dyad. To our knowledge, no other study has reported this finding before. As it is established that maternal BMI is associated with an altered infant microbiota predisposing them to obesity [87], and a higher BMI affects gut microbiota composition [88, 89], we hypothesize that specific taxa associated with BMI may be more likely transmitted to the infants, also explaining the increased similarity between infant and mother microbiota [87].

One of the strengths of this study is that we not only examined the association between the GDM status and gut microbiota composition, but we also considered variables related to overall maternal health, thereby adding complementary insights to the existing literature. Additionally, the cardiometabolic profile and maternal dietary profiles were assessed at the same time as the feces samples were collected, enabling a comprehensive investigation of the association between maternal health, diet, and gut microbiota composition in the context of GDM. Fecal samples were collected and analyzed using a standardized protocol to guarantee consistency and limit contamination sources [90]. Also, feces samples processing was blinded to research assistant, which limits information bias. Finally, our study includes both mothers and their respective infants, thus allowing us to explore the potential impact of GDM status on vertical transmission of the microbiota [91].

This study also has certain limitations. First, only one fecal sample per participant was available, which may not capture the temporal variability of the microbiota [92, 93]. Furthermore, stool samples do not fully represent the entire intestinal microbiota, as they mostly reflect bacteria from the colon, not capturing all those inhabiting the gut [94, 95]. Also, the gut microbiota composition was analyzed using 16S rRNA gene sequencing. It is well established that whole-genome shotgun sequencing offers multiple advantages over this last method, such as sequencing broader genome regions and improving bacterial species detection and gene prediction [96, 97]. Also, our small sample size reduces the statistical power and the generalizability of our findings [98]. Moreover, the small sample size may result in a sample that is not fully representative of the GDM population. For example, in our sample, only one mother in each group had a cesarean section, despite evidence indicating that cesarean deliveries are more common among mothers with GDM [99]. Since cesarean delivery has been linked to variations in infant gut microbiota composition, our findings may not accurately reflect this association [100]. Moreover, all participants were Caucasian, which reduces the generalizability of the results [101]. This limitation can be partly explained by the fact that the study was conducted in Quebec City, where the majority of the population is Caucasian, which made it challenging to recruit participants from other ethnicities [102]. However, this limitation may also be beneficial, as it helped control for gut microbiota variability that could be associated with ethnicity [103]. Additionally, due to logistical challenges in planning meetings with our participants, sample collection did not occur exactly at 2 months postpartum for all infants. At this age, microbiota is rapidly changing and is highly influenced by environmental factors [104]. Therefore, variability in the age of infants at sample collection could have affected gut microbiota composition and introduced variability in our results. However, this last possibility appears to be less likely given the link found within the mother and infant dyads, and its correlation with the mother’s BMI, or at least did not prevent us from making this novel observation.

## Conclusions

Our findings suggest that GDM exposure is associated with the gut microbiota of both mothers and infants at two months postpartum. Although the association between GDM and infant microbiota seems to diminish with time, it has been shown that gut microbiota dysbiosis in early life can still predispose individuals to various diseases later on [105]. Given the exploratory nature of this study and the small sample size, our results should be interpreted as hypothesis-generating for future research. Future studies with larger cohorts, longitudinal designs, and species-level analysis are needed to fully understand how GDM may shape the gut microbiota. Overall, our findings contribute to the growing body of evidence associating maternal health with the infant and mother gut microbiota, the full knowledge of which is needed to ultimately improve maternal and infant health outcomes.

## Data Availability

The datasets generated and/or analysed during the current study are available in the NCBI sequence read archive (SRA) repository, available at https://www.ncbi.nlm.nih.gov/sra/PRJNA1213118.

## Abbreviations

BMI: Body mass index
CDA: Canadian Diabetes Association
DEXA: Dual-energy X-ray absorptiometry
DOHaD: Developmental Origins of Health and Disease
GCT: Glucose challenge test
GDM: Gestational diabetes mellitus
GDM+: Exposition to gestational diabetes mellitus
GDM-: No exposition to gestational diabetes mellitus
GLM: Generalized linear models
HbA1c: Glycated hemoglobin
HOMA-IR: Homeostasis Model Assessment of Insulin Resistance
MFA: Multiple factor analysis
OGTT: Oral glucose tolerance test
PERMANOVA: Permutational multivariate analysis of variance
RCT: Randomized controlled trial
